# Complete meiosis in rat prepubertal testicular tissue under in vitro sequential culture conditions

**DOI:** 10.1111/andr.13325

**Published:** 2022-11-22

**Authors:** Justine Saulnier, Mathilde Soirey, Nasreddine Kébir, Marion Delessard, Aurélie Rives‐Feraille, Laura Moutard, Ludovic Dumont, Nathalie Rives, Christine Rondanino

**Affiliations:** ^1^ INSERM U1239 Adrenal and Gonadal Pathophysiology team Laboratory of Neuroendocrine Endocrine and Germinal Differentiation and Communication Rouen University Hospital Rouen Normandy University Rouen France; ^2^ Normandie Univ INSA Rouen Normandie Laboratoire PBS Saint‐Etienne‐du‐Rouvray France

**Keywords:** in vitro organ culture, meiosis, sequential condition, spermatogenesis, testis

## Abstract

**Background:**

Testicular tissue cryopreservation before gonadotoxic treatments allows fertility preservation in children suffering from cancer. Fertility restoration strategies, in particular in vitro maturation of prepubertal testicular tissue, are being developed mainly in animal models. The rat, widely used in biomedical research, including in reproductive biology, is a relevant model.

**Objectives:**

To determine whether sequential two‐step culture protocols can improve the efficiency of rat in vitro spermatogenesis.

**Materials and methods:**

Rat prepubertal testicular tissues were cultured on agarose gels with either a one‐step or two‐step protocol with or without polydimethylsiloxane (PDMS) ceiling chips. The progression of spermatogenesis, germ/Sertoli cell ratio, cell proliferation, seminiferous tubule area, and intratubular cell density were assessed by histological and immunohistochemical analyses. Terminal deoxynucleotidyl transferase‐mediated dUTP nick‐end labeling (TUNEL) assays and Peanut Agglutinin (PNA) lectin labeling were performed to analyze the Deoxyribonucleic Acid (DNA) integrity and differentiation step of in vitro‐produced spermatids.

**Results:**

Sequential two‐step protocols allowed the production of spermatids with a higher efficiency compared with the one‐step culture protocol. However, the efficiency was low, as less than 1.5% of tubules contained spermatids. Most of the in vitro‐produced spermatids contained unfragmented DNA and were at an early step of differentiation. Rare elongating spermatids could be detected in the cultured explants. Although complete in vitro spermatogenesis could not be obtained with PDMS ceiling chips, entry into meiosis was promoted in one‐step organotypic cultures.

**Discussion and conclusion:**

Complete in vitro meiosis and the beginning of the elongation phase of spermiogenesis were obtained in a rat model using sequential culture methods. Because of their low efficiency, further work will be necessary to identify the culture conditions allowing the completion of spermiogenesis. These optimizations could pave the way for future applications, including the development of an in vitro fertility restoration procedure for childhood cancer survivors, which is still far from being clinically available.

## 1 INTRODUCTION

Male fertility preservation is a process allowing the cryopreservation of germ cells before their exposure to highly gonadotoxic therapies, such as chemotherapy and radiotherapy.^1^, ^2^ Sperm freezing, which is proposed for pubertal and adult cancer patients before treatments, cannot be envisaged for young boys, who do not yet produce spermatozoa. Instead, the cryopreservation of testicular tissues containing spermatogonial stem cells (SSCs), which are particularly sensitive to cancer treatments, is the fertility preservation procedure used before treatments for these prepubertal patients.^3^ The fragments of immature testicular tissues collected during childhood are stored in the hope that they can be used in the future to restore the fertility of patients who become infertile as a result of treatments. Fertility restoration approaches, whose goal is to lead to the production of spermatozoa from immature testicular biopsies, are still at an experimental stage. SSC autotransplantation or testicular tissue autografting is potential in vivo fertility restoration approaches that cannot be proposed for patients with tumor cell contamination of their testes (approximately 30% of young boys with leukemia).^4^ Other procedures, such as in vitro maturation of SSCs^5^ or of testicular tissue (i.e., organotypic culture), are therefore being developed to allow the in vitro production of spermatozoa that could be used for assisted reproductive techniques. As testicular tissues from prepubertal patients are scarce and precious, the research in this field is conducted mainly in animal models. Organotypic culture on agarose gels is a promising approach, allowing the preservation of the SSC niche and tissue architecture as well as the production of fertilization‐competent spermatozoa from prepubertal testicular tissues in the mouse model.^6^, ^7^, ^8^, ^9^, ^10^ Recently, mouse spermatozoa have also been obtained in cultures of prepubertal testicular tissues covered by polydimethylsiloxane (PDMS) ceiling chips; this method, by flattening the tissues, avoiding their direct exposure to air, allowing optimized oxygen and nutrient supply and preventing central necrosis, improved the efficiency of mouse in vitro spermatogenesis.^11^, ^12^

The rat model is considered an advance from the mouse model toward human clinical applications^13^, ^14^ and is particularly challenging as complete in vitro spermatogenesis has not yet been achieved in this species. Attempts of in vitro maturation of prepubertal testicular tissues from rats, whose spermatogenesis duration is longer than in mice (53 days vs. 35 days), were performed on agarose gels and led to the production of spermatocytes I^15^, ^16^ or few round spermatids.^13^, ^14^ The use of polydimethylsiloxane (PDMS) ceiling chips significantly but modestly promoted rat in vitro spermatogenesis.^14^ Optimizing organotypic culture protocols in this animal model could help us progress in the development of an in vitro fertility restoration procedure. In the studies mentioned above,^13^, ^14^, ^15^ in vitro cultures were performed with the same medium throughout the culture. However, spermatogenesis is a complex process precisely regulated by the sequential appearance of different factors. We previously compared the efficiency of rat in vitro spermatogenesis using six different culture media.^15^ After 16 days of culture, up to 53% of seminiferous tubules containing spermatocytes I were observed when DMEM/F12 medium supplemented with knockout serum replacement (KSR), vitamins A, C and E, retinoic acid, follicle‐stimulating hormone, testosterone, insulin, apo‐transferrin and pyruvic acid (so‐called “supplemented medium [SM] medium” in the present study) was used.^15^ However, spermatocytes I did not survive at later time points under this culture condition.^15^ Conversely, the use of α‐minimum essential medium (α‐MEM) medium supplemented with KSR and vitamins A and E (so‐called “basal medium [BM] medium” in the present study) allowed the longer‐term maintenance of spermatocytes I in organotypic cultures: approximately 12% and 34% of tubules contained these germ cells after 16 days and 45 days, respectively.^15^ Based on these observations, we hypothesized that the progression of rat in vitro spermatogenesis and germ cell survival could be promoted by applying a sequential two‐step culture protocol.

In the present study, rat prepubertal testicular tissues were therefore either cultured with a one‐step or two‐step protocol with or without PDMS ceiling chips. The impact of these culture conditions on the progression of in vitro spermatogenesis was compared.

## 2 MATERIALS AND METHODS

### 2.1 Animal care and handling

All experimental procedures were approved by the Institutional Animal Care and Use Committee of Rouen Normandy University under license/protocol number: 26016–2020061209299793. Wistar rats were housed in a temperature‐ and humidity‐controlled environment under a 12‐h light/dark cycle. Food and water were provided ad libitum. Pups aged 8.5 d*pp* were euthanized by decapitation.

The Wistar strain was chosen for the following reasons: (i) we previously used this strain to assess freezing procedures on immature testicular tissues, to compare the efficiency of in vitro spermatogenesis under various culture conditions and to identify differentially expressed genes between in vitro and in vivo conditions using RNA‐sequencing,^15^, ^16^, ^17^ (ii) this strain was previously used by another team to investigate in vitro spermatogenesis, with a blockage at the pachytene spermatocyte stage,^18^ and (iii) Wistar rats are used for toxicology studies and for biomedical research.

### 2.2 Study design

**FIGURE 1:**
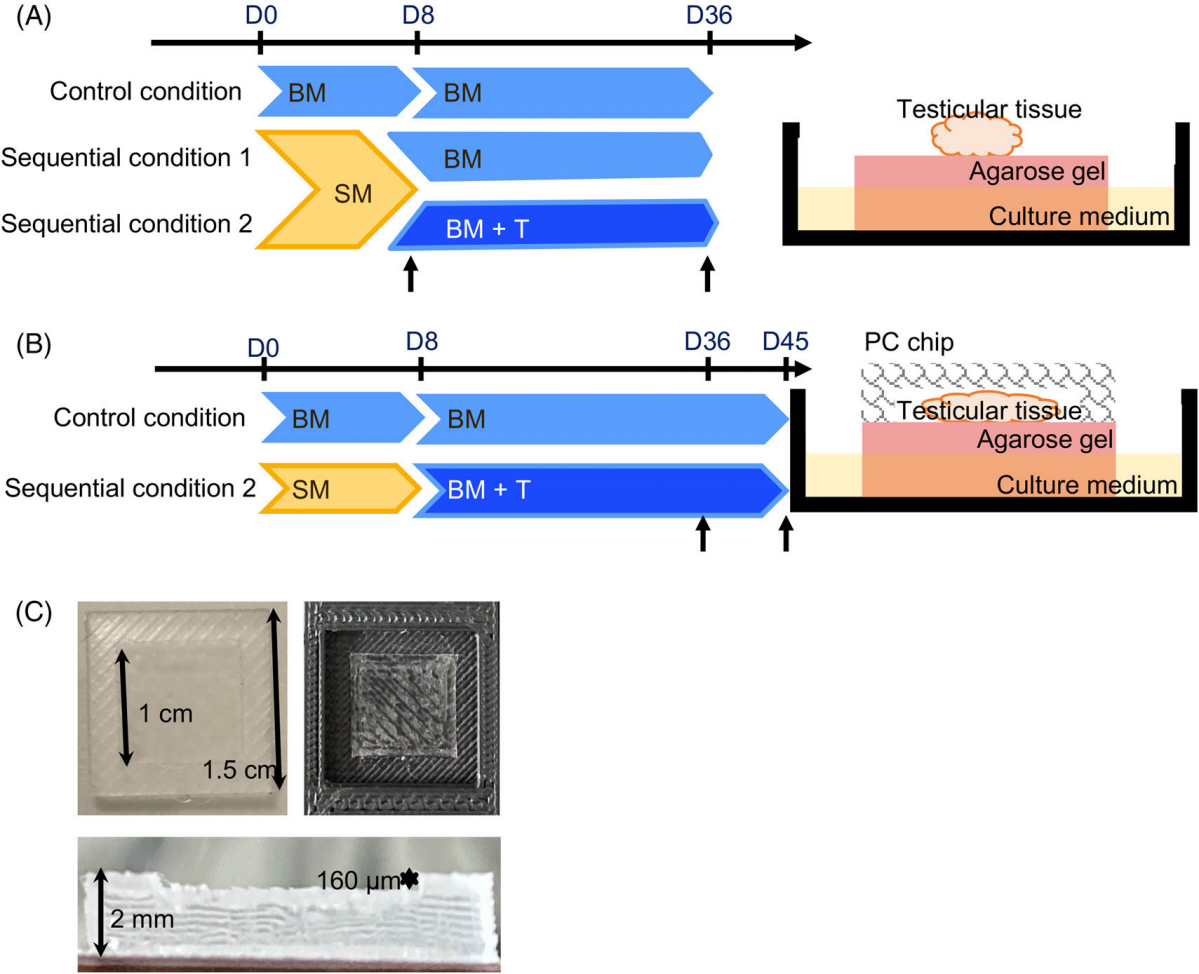
Flow chart of the study design and organotypic culture conditions. (A) Rat prepubertal testicular tissues were cultured at a gas‒liquid interphase on agarose gels until D36. The control condition was a one‐step culture with basal medium (BM) medium. The Sequential 1 and 2 conditions are two‐step cultures: cultures with supplemented medium (SM) medium until D8 followed by BM medium (sequential condition 1) or BM medium supplemented with testosterone (sequential condition 2) until D36. Arrows indicate culture arrests at D8, D36, or D45 for tissue analyses. (B) Rat prepubertal testicular tissues, flattened under PC chips, were cultured on agarose gels until D36 or D45 using either the control or Sequential 2 condition. For each experiment, 12 testicular fragments were analyzed. (C) Pictures of the PC chip, top view on the left, side view below and one cavity of the 3D‐printed master mold on the right. The PC chip has a square cavity of 1 cm^2^ with a depth of 160 µm. BM(+T), basal medium (+testosterone); D, day; PC chip, polydimethylsiloxane ceiling chip; SM, supplemented medium

Three different culture conditions, called the “control condition” (Ctrl), “Sequential condition 1” (Seq1), and “Sequential condition 2” (Seq2), were tested in this study (Figure 1A). Sequential culture protocols comprise two steps: (i) an 8‐day culture with SM (Table 1) followed by a 28‐day or 37‐day culture with BM or BM containing testosterone (BM+T) (Table 1). SM medium was chosen for the first phase of the cultures because it is efficient in promoting meiotic entry.^15^ However, spermatocytes I did not survive with this medium beyond 16 days of culture.^15^ Conversely, BM medium was less efficient in promoting entry into meiosis but was the best medium (among the six media previously tested), allowing the survival of spermatocytes I up to 45 days of culture.^15^ It was therefore chosen for the second step of sequential cultures. As testosterone is known to promote meiosis completion and spermiogenesis, BM+T medium was also tested in this study. To promote meiotic entry while minimizing depletion in spermatocytes I, we chose to switch from SM medium to BM or BM+T medium after 8 days of culture. The control condition is a one‐step culture with BM, which allows better survival of spermatocytes I as previously described.^15^ Cultures of testicular tissue explants underneath a PDMS ceiling chip were also performed with BM or the best sequential culture protocol (Figure 1B,C). For each experiment, 12 testicular fragments were cultured, and up to 60 cross‐sectioned seminiferous tubules (from two sections separated by at least 30 µm) were analyzed in each fragment. The total number of seminiferous tubules analyzed is shown in Supporting Information S1.

**TABLE 1:** Composition of the media used in the sequential culture method

Culture media	BM (+T)^a^	SM^b^
**Medium**	α‐MEM	DMEM/F12
**KSR**	10%	10%
**Vitamin C**	/	0.1 mM
**Vitamin E**	3400 µM	23.2 µM
**Retinol**	1 µM	0.33 µM
**Retinoic acid**	/	0.33 µM
**FSH**	/	50 ng/ml
**Testosterone**	/ (+0.1 µM)	0.1 µM
**Insulin**	/	10 µg/ml
**Apo‐transferrin**	/	10 µg/ml
**Pyruvic acid**	/	1 mM
**Antibiotic**	Gentamicin	Gentamicin + P/S

*Note*: Testes from 8.5 d*pp* rats were cultured until D8 with either BM or SM media and until D36 or D45 with BM medium with (BM+T) or without testosterone supplementation.

Abbreviations: α‐MEM, α‐minimum essential medium; BM, basal medium; DMEM/F12, Dulbecco's modified Eagle's medium/nutrient mixture F‐12; d*pp*, days *postpartum*; FSH, follicle‐stimulating hormone; KSR, knockout serum replacement; P/S, penicillin/streptomycin; SM, supplemented medium; T, testosterone.

^a^ Medium B.^15^

^b^ Medium D.^15^ ^.^

### 2.3 Organotypic cultures

In the present study, we chose to culture 8.5 d*pp* rat testicular tissues for the following reasons: (i) they contain spermatogonia while gonocytes are no longer present, and the mitotic activity of Sertoli cells is very low,^19^ that is, their germ cell content is close to that found in testicular tissue samples from prepubertal boys who could benefit from in vitro spermatogenesis; and (ii) we previously found in both the mouse and rat models that in vitro germ cell differentiation was more efficient when prepubertal testes contained spermatogonia rather than gonocytes.^8^

Testicular explants were cultured using the gas‒liquid interphase method as previously described.^15^, ^16^ The day before the beginning of each culture, 1.5% agarose gels were soaked completely in medium (αMEM + 0.01% gentamicin) and kept overnight in the incubator. Prepubertal testes from rats aged 8.5 d*pp* were cut into six fragments and cultured onto agarose gels (Sigma‒Aldrich, Saint‐Quentin‐Fallavier, France) at 34°C under 5% CO_2_ in air. Culture media were prepared and replaced every 3–4 days.

### 2.4 PDMS ceiling chips

Several studies have demonstrated that the addition of PDMS ceiling chips on testicular tissues promotes in vitro spermatogenesis.^12^, ^14^, ^20^ We report here for the first time the making of a 3D‐printed master mold designed to produce PDMS chips quickly and in large quantities. The master mold with 25 square cavities (size: 1.5 × 1.5 × 0.18 cm^3^) containing a square central block (size: 1 × 1 × 0.016 cm^3^) was obtained with a 3D printer (Tiertime, UpBox) using a thermoplastic copolymer, namely acrylonitrile butadiene styrene as the printing material (Supporting Information S2 and S3). The 3D printing process resulted in grooved surfaces. The surface of the central blocks was smoothed with paper glue and then covered with thin tape (thickness: 0.003 cm) (Figure 1C). Then, a mixture of the two components of Sylgard 184 (DOW Chemical Company, Villers‐Saint‐Paul, France), that is, base and hardener in a weight ratio of 10:1 was poured into the 3D printed master mold. The mixture was then degassed under vacuum until air bubbles were completely removed. The PDMS was then crosslinked at room temperature (to avoid thermal deformation of the master mold) for 2 days. Finally, the individual chips (Figure 1C) were peeled off the master mold using a knife cutter.

### 2.5 Histological and immunohistochemical analyses

Cultured explants were fixed for 2 h at room temperature in Bouin's solution for histological analyses or in 4% paraformaldehyde for immunofluorescence and terminal deoxynucleotidyl transferase‐mediated dUTP nick‐end labeling (TUNEL) analyses. They were dehydrated in successive ethanol/xylene baths in the Citadel 2000 tissue processor (Shandon, Cheshire, UK) and embedded in paraffin. Tissue sections (3 µm thick) were cut with the JungRM 2015 microtome (Leica Microsystems GmbH, Wetzlar, Germany) and mounted on Polysine slides (Thermo Fisher Scientific, Saint‐Aubin, France). Tissue sections were then deparaffinized in xylene and rehydrated with decreasing concentrations of ethanol.

***Hematoxylin‐eosin‐saffron (HES) staining and periodic acid Schiff (PAS) reaction***. The area of seminiferous tubules was assessed on HES‐stained tissue sections. The progression of spermatogenesis and intratubular cell density (number of intratubular cells/1000 µm^2^) were analyzed on PAS‐stained (RAL diagnostic, Martillac, France) tissue sections. The identification of the different types of germ cells was based on the morphological aspect of their nucleus and their localization in the seminiferous tubules.^21^ Moreover, the PAS reaction allowed the visualization of the acrosomal cap of spermatids. Cross‐sectioned seminiferous tubules, whose ratio between the longest diameter and its perpendicular diameter was below 1.5, were analyzed. Cultured testicular fragments were analyzed with a light microscope (DM4000B, Leica Microsystems GmbH) equipped with a digital Leica Application Suite imaging analysis system.

***Ki67 and DDX4 immunofluorescence staining***. Cell proliferation and the germ cell/Sertoli cell ratio were assessed in Ki67‐ and DDX4‐immunostained tissue sections, respectively, as previously described.^15^ Briefly, tissue sections were incubated in citrate buffer (Diapath, Martinengo, Italy) at 96°C for 40 min and cooled for 20 min at RT. After rinsing in distilled water, sections were permeabilized with 0.01% Triton X‐100 in Phosphate‐Buffered Saline (PBS) for 15 min at RT (for Ki67 immunostaining only). To avoid nonspecific antibody binding, sections were blocked with 5% BSA and 5% horse serum in PBS (for Ki67) or with 5% BSA and 20% goat serum in PBS (for DDX4). Tissue sections were then incubated overnight with anti‐Ki67 (1:500, ab16667, Abcam, Cambridge, UK) or anti‐DDX4 (1:800, ab13840, Abcam) primary antibodies at 4°C. Negative controls were performed by omitting the primary antibody. After three washes in PBS containing 0.05% Tween‐20, the sections were incubated with goat antirabbit secondary antibodies coupled to Alexa Fluor 488 (1:200, ab150077, Abcam) for 1 h at RT. The sections were rinsed, dehydrated with ethanol, and mounted in Vectashield (Vector, Eurobio, Les Ulis, France) with Hoechst 33342 (Thermo Fisher Scientific) to counterstain cell nuclei. Analyses were conducted with an epifluorescence microscope (DMRBE, Leica Microsystems GmbH) equipped with a monochrome CCD IEEE1394 FireWire video camera (Perceptive Instruments, Bury St Edmunds, UK).

### 2.6 TUNEL analyses and PNA lectin labeling

TUNEL assays were performed using the In Situ Cell Death Kit POD, following the manufacturer's instructions (Roche, Mannheim, Germany). Then, the sections were postfixed with 4% paraformaldehyde (PFA) for 15 min at RT, incubated in citrate buffer for 20 min and blocked with 5% BSA and 5% horse serum in PBS. The sections were then incubated with Peanut Agglutinin (PNA) lectin conjugated to Alexa Fluor 594 (1:200, L32459, Thermo Fisher Scientific) for 30 min at RT to reveal the acrosome of spermatids and counterstained with Hoechst 33342. Analyses were conducted with an epifluorescence microscope as described above. Spermatid differentiation was evaluated as described by Leblond and Clermont.^22^ As individual steps were hardly identifiable on cross sections, spermatids were classified into three categories based on the shapes of the nucleus and/or the acrosome: steps 1–7 for round spermatids (round nucleus), steps 8–11 for elongating spermatids (elongating nucleus and acrosome) and steps 12–19 for elongated spermatids (thinner nucleus and hook‐shaped nucleus).

### 2.7 Statistical analyses

Statistical analyses were performed with GraphPad Prism v6.0 software (GraphPad Software Inc., La Jolla, CA, USA). Nonparametric Mann‒Whitney tests, Chi^2^ tests and Kruskal‒Wallis tests followed by Dunn's multiple comparison were performed as specified in each figure. A value of *p* <0.05 was considered statistically significant.

## 3 RESULTS

### 3.1 Germ cell differentiation is promoted in short‐term organotypic cultures with SM medium

**FIGURE 2:**
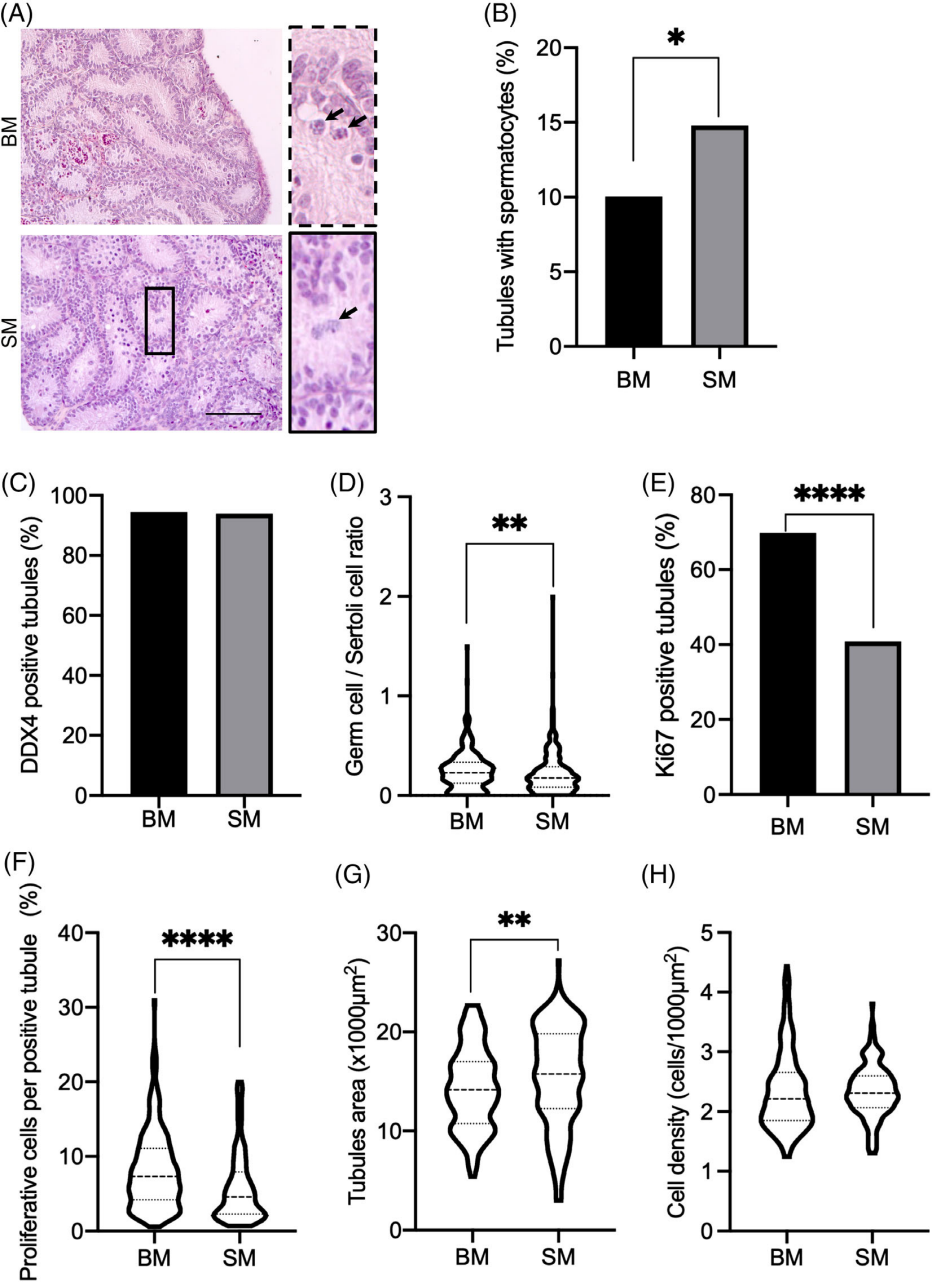
Histological analyses of rat testicular tissues at D8. (A) Representative microscopy images of HES‐stained rat testicular tissues cultured with either basal medium (BM) or supplemented medium (SM). The images on the right show magnifications of testicular tissues cultured with BM (dashed lines) or SM (solid lines). Black arrows show spermatocytes I. Magnification: ×200, scale bar: 100 μm. (B) Percentage of seminiferous tubules with at least one spermatocyte I. (C) Percentage of seminiferous tubules containing germ cells. (D) Germ cell/Sertoli cell ratio. (E) Percentage of seminiferous tubules containing proliferating cells. (F) Percentage of proliferating cells per Ki67+ tubule. (G) Area of seminiferous tubules. (H) Intratubular cell density. In violin plots, the dashed line represents the mean and the dotted lines represent the upper (Q3) and lower (Q1) quartiles. For each experiment, 12 testicular fragments were analyzed, with 575 ± 66, 386 ± 34, 591 ± 143, and 180 ± 0 seminiferous tubules analyzed per condition (mean ± s.d.) for the analyses of spermatogenesis progression, germ cell content, cell proliferation and morphometry, respectively. For statistical analyses, the chi^2^ test was applied for the B, C, and E histograms, and the Mann‒Whitney test was applied for the D, F, G, and H plots. A *p* value <0.05 was considered statistically significant. *p < 0.05, **p < 0.01, and ****p < 0.0001. BM, basal medium; D, day; HES, hematoxylin‐eosin‐saffron; s.d., standard deviation; SM, supplemented medium

Rat prepubertal testicular tissues were first cultured for 8 days with either BM or SM (Figure 1A). Spermatocytes I were the most differentiated germ cells observed in seminiferous tubules under both culture conditions (Figure 2A). A higher proportion of tubules with spermatocytes I was found when using SM rather than BM (14.8% vs. 10%, *p* = 0.015, Figure 2B). However, the percentage of tubules containing germ cells was similar between the two conditions (Figure 2C). Moreover, a lower ratio of germ cells to Sertoli cells, a lower percentage of tubules containing proliferating cells and a lower percentage of proliferating cells per tubule were observed with SM than with BM (0.22 vs. 0.24, *p* = 0.0021, Figure 2D; 40.8 vs. 69.8%, *p* < 0.0001, Figure 2E; 5.80 vs. 8.16%, *p* < 0.0001, Figure 2F). While the area of seminiferous tubules was higher after organotypic culture with SM than BM (15551 vs. 14326 µm^2^, *p* = 0.0047, Figure 2G), no difference in intratubular cell density was found between the two culture conditions (Figure 2H).

### 3.2 Spermatid production is promoted in 36‐day organotypic cultures with the Seq2 protocol

**FIGURE 3:**
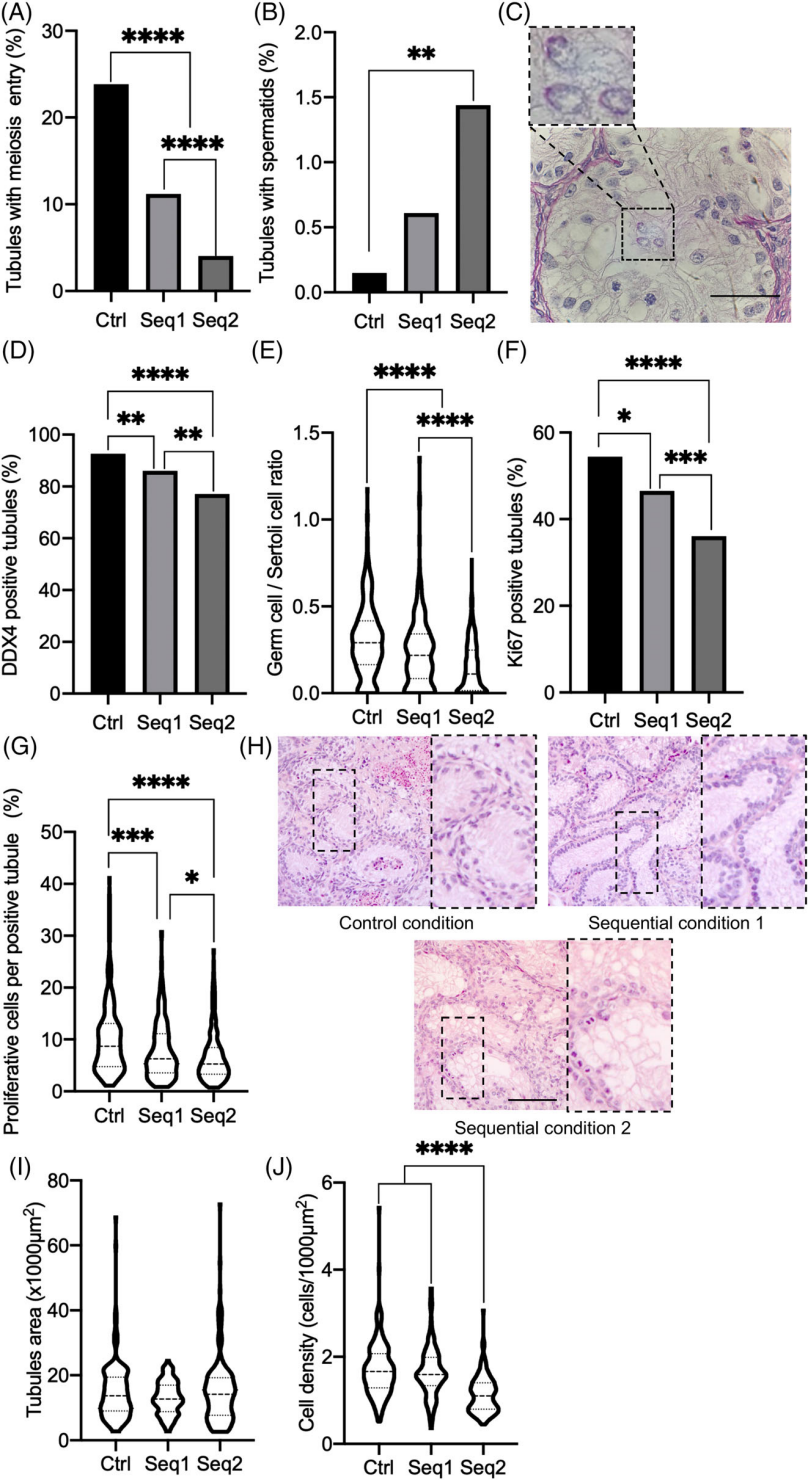
Histological analyses of rat testicular tissues at D36 after one‐step or two‐step cultures. (A) Percentage of seminiferous tubules containing at least one meiotic cell. (B) Percentage of seminiferous tubules containing at least one spermatid. (C) Representative microscopy image of a seminiferous tubule stained with PAS, revealing the presence of in vitro‐produced spermatids. The upper insert is a magnified image showing the pink‐colored acrosomes of spermatids. Magnification: ×500, scale bar: 50 μm. (D) Percentage of seminiferous tubules containing germ cells. (E) Germ cell/Sertoli cell ratio. (F) Percentage of seminiferous tubules containing proliferating cells. (G) Percentage of proliferating cells per Ki67+ tubule. (H) Representative microscopy images of cultured rat testicular tissues stained with HES. Inserts show magnified images of the selected areas in dotted lines. Magnification: ×200, scale bar: 50 μm. (I) Area of seminiferous tubules. (J) Intratubular cell density. In violin plots, the dashed line represents the mean, and the dotted lines represent the upper (Q3) and lower (Q1) quartiles. For each experiment, 12 testicular fragments were analyzed, with 664 ± 25, 334 ± 48, 521 ± 41, and 180 ± 0 seminiferous tubules analyzed per condition (mean ± s.d.) for the analyses of spermatogenesis progression, germ cell content, cell proliferation, and morphometry, respectively. For statistical analyses, the chi^2^ test was applied for the A, B, D, and F histograms, and the Kruskal‒Wallis test followed by Dunn's posttest was applied for the E, G, I, and J plots. A *p* value <0.05 was considered statistically significant. *p < 0.05, **p < 0.01, ***p < 0.001, and ****p < 0.0001. Ctrl, control condition; D, day; HES, hematoxylin‐eosin‐saffron; PAS, periodic acid Schiff; s.d., standard deviation; Seq1/2, Sequential condition 1/2

Rat prepubertal testicular tissues were then cultured for 36 days with a one‐step protocol (BM only: Ctrl condition) or sequential two‐step protocols (SM/BM for Seq1 or SM/BM+T for Seq2) (Figure 1A). Entry into meiosis was observed in all culture conditions (Figure 3A). A higher percentage of seminiferous tubules with meiotic cells was obtained with the Ctrl condition than with the Seq1 or Seq2 protocols (23.8% vs. 11.2% or 4.04%, *p* < 0.0001, Figure 3A). Rat in vitro spermatogenesis reached the spermatid stage at this time point (Figure 3B,C). Interestingly, a significantly higher proportion of tubules contained spermatids with the Seq2 condition than with the Ctrl protocol (1.44% vs. 0.15%, *p* < 0.0091, Figure 3B). However, the percentage of tubules containing germ cells and the ratio of germ cells to Sertoli cells were significantly lower when using Seq2 than when using Ctrl or Seq1 (77.2 vs. 92.6 or 86.0%, *p* < 0.0001 or *p* = 0.0046, Figure 3D; 0.15 vs. 0.30 or 0.23, *p* < 0.0001, Figure 3E). Moreover, significantly lower proportions of tubules containing proliferating cells and of proliferating cells per tubule were found after Seq2 cultures than after Ctrl or Seq1 cultures (36.1% vs. 54.4% or 46.5%, *p* < 0.0001 or *p* = 0.0123, Figure 3F; 6.67% vs. 10.2% or 7.88%, *p* < 0.0001 or *p* < 0.05, Figure 3G). Similar tubular areas were observed between the different culture conditions (Figure 3H,I). The intratubular cell density was however lower after Seq2 organotypic cultures than after Ctrl or Seq1 cultures (1.13 cells/1000 μm^2^ vs. 1.71 or 1.65 cells/1000 μm^2^, *p* < 0.0001, Figure 3J).

### 3.3 Most in vitro‐produced spermatids contain unfragmented DNA and are at an early step of differentiation

**FIGURE 4:**
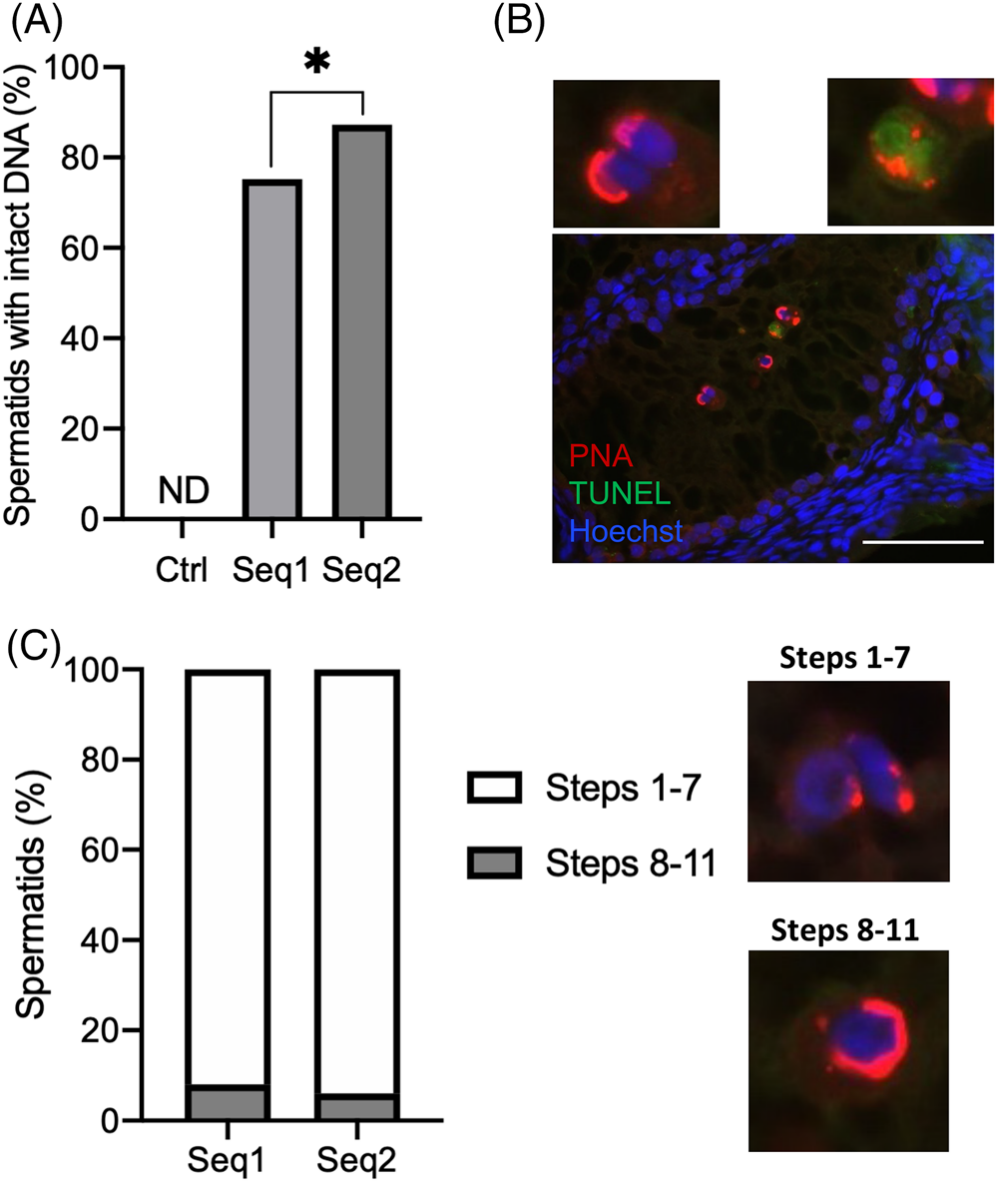
DNA integrity and differentiation step of in vitro‐produced spermatids. (A) Percentage of spermatids with intact DNA (TUNEL negative) obtained under the different culture conditions. No spermatids were found in testicular fragments cultured under the Ctrl culture condition. (B) Representative fluorescence microscopy image of cultured rat testicular tissues stained with PNA lectin for spermatid acrosome (red), TUNEL for DNA fragmentation (green) and Hoechst for cell nucleus (blue). The upper left magnification shows spermatids with intact DNA (TUNEL negative), and the upper right magnification shows a spermatid with fragmented DNA (TUNEL positive). Magnification: ×960, scale bar: 50 μm. (C) Percentage of spermatids at steps 1–7 (white bar) and 8–11 (gray bar) of differentiation. Images on the right illustrate these steps, distinguishable by their PNA lectin labeling patterns and the shape of their nuclei. For each experiment, 12 testicular fragments were analyzed, with 95 ± 114 cells analyzed per condition (mean ± s.d.). For statistical analyses, the chi^2^ test was applied. A *p* value <0.05 was considered statistically significant. *p < 0.05. Ctrl, control condition; D, day; DNA, Deoxyribonucleic Acid; ND, no data; PNA, Peanut Agglutinin; s.d., standard deviation; Seq1/2, Sequential condition 1/2; TUNEL, terminal deoxynucleotidyl transferase‐mediated dUTP nick‐end labeling

Although rare spermatids could be observed in the Bouin's fixed and PAS‐stained Ctrl tissue sections that were analyzed (Figure 3B), no spermatids were detected in the PFA‐fixed and PNA‐labeled Ctrl tissue sections that were analyzed (Figure 4A). In contrast, spermatids were found for the Seq1 and Seq2 conditions in both the PAS‐stained and PNA‐labeled tissue sections that were analyzed (Figures 3B and 4A).

The DNA (Deoxyribonucleic acid) integrity and differentiation step of spermatids generated in vitro with the sequential two‐step protocols were assessed by TUNEL analyses and PNA lectin labeling. A higher percentage of spermatids with intact DNA was found under Seq2 than under Seq1 (87.3% vs. 75.2%, *p* = 0.0292, Figure 4A,B). Step 1–7 spermatids were mainly observed in testicular explants (92% or 94% for Seq1 or Seq2, respectively, Figure 4C). Step 8–11 spermatids were present in lesser proportions (8% or 6% for Seq1 or Seq2, respectively, Figure 4C). The mean number of spermatids per tubule was 5.16 ± 3.56 and 3.71 ± 2.64 (mean ± s.d.) with the Seq1 and Seq2 conditions, respectively. No spermatids at steps 12–19 of differentiation were obtained with the Seq1 or Seq2 protocol.

### 3.4 The efficiency of rat in vitro spermatogenesis is not enhanced under the PDMS ceiling chip

**FIGURE 5:**
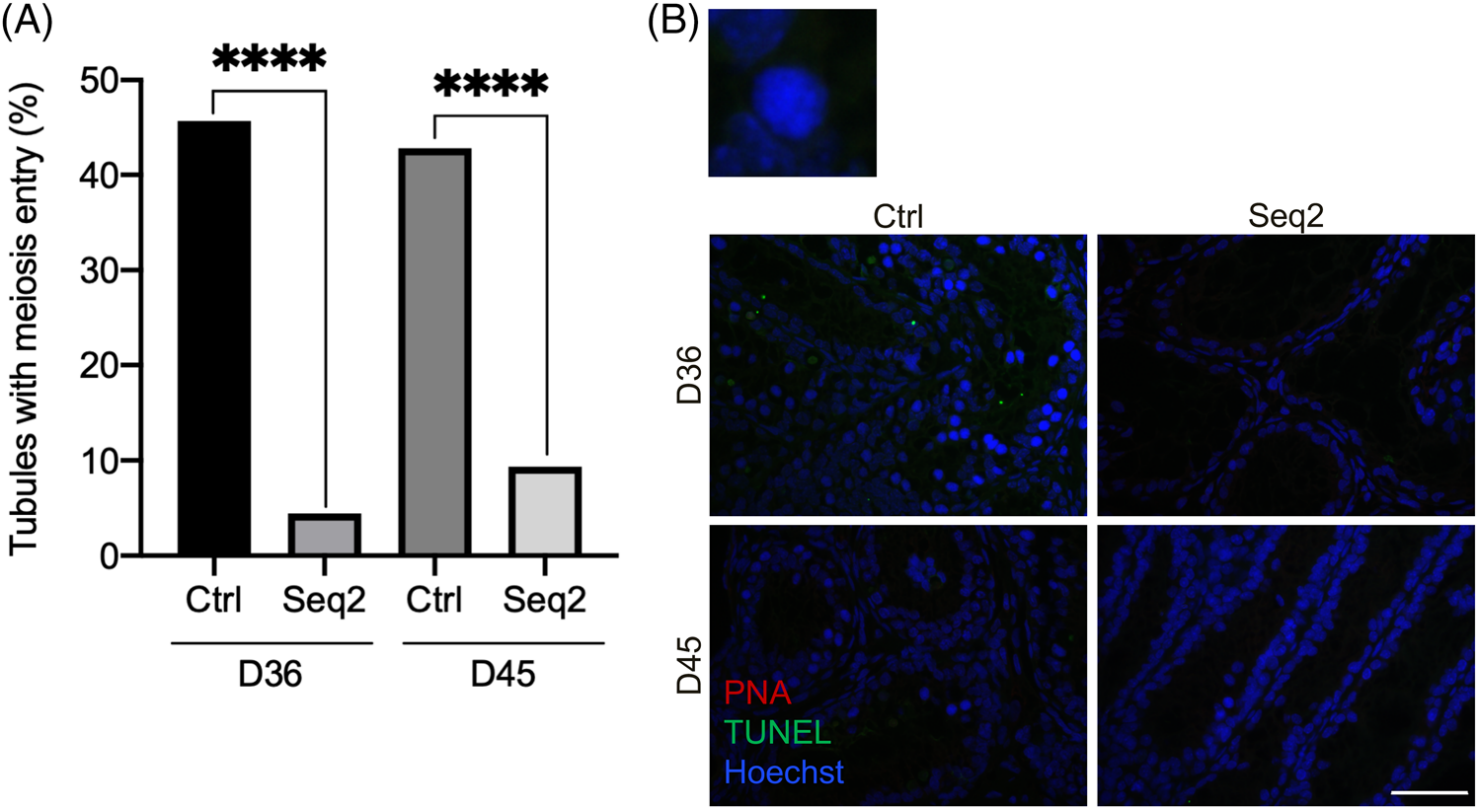
Analyses of rat testicular tissues cultured with polydimethylsiloxane (PDMS) ceiling chips until D36 and D45. (A) Percentage of seminiferous tubules containing at least one meiotic stage in rat testicular tissues cultured until D36 or D45. For each experiment, 12 testicular fragments were analyzed, with 228 ± 37 and 184 ± 63 seminiferous tubules analyzed per condition (mean ± s.d.) at D36 and D45, respectively. For statistical analyses, chi^2^ test was applied. A *p* value <0.05 was considered statistically significant. ****p < 0.0001. (B) Representative fluorescence microscopy images of cultured rat testicular tissues stained with PNA lectin for spermatid acrosome (red), TUNEL for DNA fragmentation (green) and Hoechst for cell nucleus (blue). The upper left magnification shows a representative spermatocyte I. Magnification: ×400, scale bar: 50 μm. Ctrl, control condition; D, day; DNA, Deoxyribonucleic Acid; PNA, Peanut Agglutinin; Seq2, Sequential condition 2; TUNEL, terminal deoxynucleotidyl transferase‐mediated dUTP nick‐end labeling.

Rat prepubertal testicular tissues were then cultured for up to 45 days (i.e., until the end of the first spermatogenic wave) either under the Ctrl, or the best sequential culture condition (Seq2) to assess whether complete spermatogenesis can be obtained (Figure 1B). Moreover, with the aim of increasing the efficiency of rat in vitro spermatogenesis, organotypic cultures were performed with PDMS ceiling chips (Figure 1B,C). At D36, the percentage of seminiferous tubules containing spermatocytes was significantly higher when using PDMS ceiling chips in the Ctrl condition (46.1% vs. 23.8%, *p* < 0.0001, Figures 3A and 5) but not in the Seq2 condition. In 36‐day and 45‐day cultures with PDMS ceiling chips, the percentage of tubules with meiotic cells was higher in Ctrl than in Seq2 conditions (45.67 vs. 4.46% and 42.79 vs. 9.35%, *p* < 0.0001, Figure 5). No spermatids were found at D36 or D45 in organotypic cultures under the PDMS ceiling chip regardless of the culture protocol used (Figure 5).

## 4 DISCUSSION

In this study, two‐step organotypic culture protocols were used for the in vitro maturation of rat prepubertal testicular tissues with the aim of improving the efficiency of in vitro spermatogenesis. Sequential protocols could indeed better mimic physiological conditions, during which different factors regulating spermatogenesis intervene at different steps. Optimized in vitro spermatogenesis procedures could later have several potential human applications in the clinic, including fertility restoration in childhood cancer survivors.

We previously found a differentiation of rat germ cells up to the spermatocyte I stage in organotypic cultures performed at a gas‒liquid interphase using six different culture media, including SM and BM.^15^ We hypothesized that SM promotes rapid entry into meiosis, while BM allows the maintenance of spermatocytes I in cultured explants for up to 45 days.^15^ In the present work, an increased percentage of seminiferous tubules containing spermatocytes I was indeed found after an 8‐day culture in the presence of SM. Moreover, the decreased cell proliferation obtained with SM shows that the proliferation/differentiation balance was in favor of differentiation with this culture medium. In addition, we found in this study that SM promoted seminiferous tubule growth.

Following this first phase of culture, BM or BM+T medium, which contains KSR and vitamin A ± testosterone, was added to promote both germ cell survival and differentiation. Sequential two‐step protocols allowed the production of spermatids with a higher efficiency compared with our one‐step culture protocol. However, the efficiency of sequential organotypic cultures was low, as less than 1.5% of tubules contained spermatids. To date, two studies have reported the in vitro production of round spermatids, albeit with a low efficiency, in rat prepubertal testicular tissues cultured at a gas‒liquid interphase using a single culture medium^13^, ^14^; indeed, up to 4% of the tubules contained round spermatids in the former and up to 1% in the latter when using 20% O_2_. Up to 12%–13% of tubules with round spermatids were recently observed when cultures were carried out under 15% O_2_.^14^ The proportion of tubules containing spermatids was not significantly increased when we added testosterone during the second phase of sequential cultures. However, the addition of testosterone may have a negative impact on spermatogenesis as the percentage of tubules containing germ cells; the germ cell/Sertoli cell ratio and cell proliferation were lower in Seq2 than in Seq1 cultures. Other concentrations of T need to be tested in future studies to further optimize germ cell differentiation in organotypic cultures.

Moreover, we show that most of the spermatids produced in sequential cultures contained intact DNA. The analysis of the shapes of the nucleus and/or the acrosome revealed that spermatids up to steps 8–11 of their differentiation could be obtained, corresponding to the beginning of the elongation phase of rat spermiogenesis.^22^ The round and elongating spermatids detected here at D36 have been previously observed at the corresponding in vivo time frame,^23^ which indicates that rat in vitro spermatogenesis progressed at the same pace as in vivo spermatogenesis. However, the completion of in vitro spermiogenesis is a limiting step in the rat model, as previously shown under other culture conditions.^13^, ^14^ Further work will be necessary to identify the mechanisms blocking the progression of spermiogenesis and the critical factors needed to overcome this step.

It has been previously reported that the central area of testicular explants cultured at a gas‒liquid interphase suffers from oxygen deficiency and a low nutrient supply, leading to the formation of a central necrotic zone.^6^, ^24^ PDMS ceiling chips were used to flatten testicular tissue fragments onto agarose gels to successfully prevent their central necrosis.^11^, ^12^ Covering testicular explants with PDMS ceiling chips also prevents their direct exposure to air, as oxidative stress negatively impacts the progression of in vitro spermatogenesis.^24^ Promisingly, we found meiotic cells in a greater proportion of tubules in our one‐step cultures covered by PDMS ceiling chips. This is in agreement with a previous study showing in the mouse model that the PDMS ceiling chip method yielded more numerous meiotic germ cells.^12^ However, in contrast to a recent study performed in a rat model,^14^ we could not detect any spermatids in testicular explants when PDMS ceiling chips were used. In this latter study, however, a higher production of spermatids was obtained when cultures were performed without PDMS ceiling chips.^14^ As the diffusion of nutrients is facilitated by PDMS ceiling chips, the concentrations of culture media components must be adapted for flattened testicular explants. An excessive concentration of testosterone could for instance lead to increased germ cell apoptosis.^25^

In conclusion, this study reports the completion of rat in vitro meiosis under sequential two‐step conditions, with the generation of round and elongating spermatids. Further optimization of the culture conditions is required to increase the spermatid yield and overcome the arrest in spermatid development. The achievement of complete in vitro spermatogenesis in the rat model could pave the way for future applications, including the development of a fertility restoration procedure for pediatric cancer survivors.

## AUTHOR CONTRIBUTIONS

JS conceived, designed, and performed the experiments, acquired, analyzed, and interpreted the data, and drafted the manuscript. MS assisted in the acquisition of data and analyzed and interpreted the data. NK conceived a 3D printed master mold and produced PDMS ceiling chips. MD, ARF, LM, and LD reviewed the manuscript. NR and CR supervised the project and corrected the manuscript. All of the authors approved the final version of the manuscript.

## CONFLICT OF INTEREST

The authors declare that there is no conflict of interest that could be perceived as prejudicing the impartiality of the research reported.

## Supporting information

Supporting Information

Supporting Information

## Data Availability

The data that support the findings of this study are available from the corresponding author upon reasonable request.
