# Supplementary material for: Complete meiosis in rat prepubertal testicular tissue under in vitro sequential culture conditions
Source: Andrology. 2022 Nov 22;11(1):167–76. doi: 10.1111/andr.13325 (PMC10099474; doi:10.1111/andr.13325)
Supplement: Supplementary file 1 — Supporting Information [file ANDR-11-167-s001.docx]

| **Culture time point** | **PDMS ceiling chip** | **Analyses** | **Number of cultured fragments** | **Number of tubules analyzed per condition**  **(mean ± s.d.)** | **Total number of tubules analyzed** |
| --- | --- | --- | --- | --- | --- |
| D8 | Without | Most advanced stage of spermatogenesis (HES) | 12 | 575 ± 66 | 1151 |
|  |  | Germ cell content (DDX4) | 12 | 386 ± 34 | 772 |
|  |  | Cell proliferation (Ki67) | 12 | 591 ± 143 | 1183 |
|  |  | Morphometric analyses | 12 | 180 ± 0 | 360 |
| D36 |  | Most advanced stage of spermatogenesis (PAS) | 12 | 664 ± 25 | 1992 |
|  |  | Germ cell content (DDX4) | 12 | 334 ± 48 | 1003 |
|  |  | Cell proliferation (Ki67) | 12 | 521 ± 41 | 1562 |
|  |  | Morphometric analyses | 12 | 180 ± 0 | 540 |
|  |  | Spermatid differentiation and DNA integrity* | 12 | 95 ± 114* | 285* |
|  | With | Most advanced stage of spermatogenesis | 12 | 228 ± 37 | 456 |
| D45 |  | Most advanced stage of spermatogenesis | 12 | 184 ± 63 | 368 |

**Supporting information 1: Experimental conditions and analyses performed in this study**

PAS, periodic acid Schiff

* Expressed in “Number of cells analyzed per condition (mean ± s.d.)” and “Total number of analyzed cells”.
