# Supplementary material for: Complete meiosis in rat prepubertal testicular tissue under in vitro sequential culture conditions
Source: Andrology. 2022 Nov 22;11(1):167–76. doi: 10.1111/andr.13325 (PMC10099474; doi:10.1111/andr.13325)
Supplement: Supplementary file 2 — Supporting Information [file ANDR-11-167-s002.pdf]

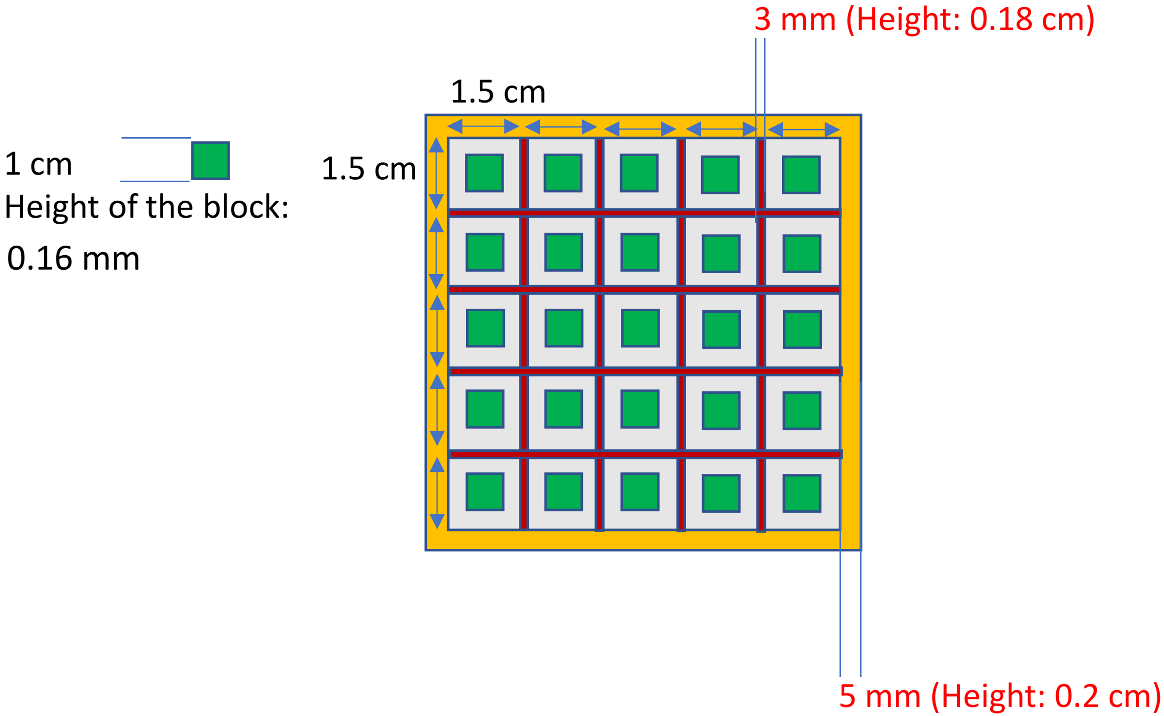

Supporting information 2: Scheme of a master mold with 25 cavities (top view not to scale).

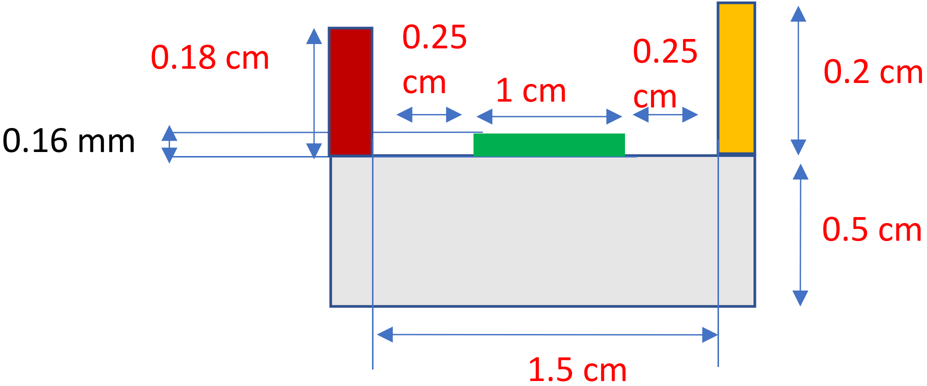

Supporting information 3: A profile cut of a cavity next to the edge (not to scale).
